# Third- and Late Line Treatments of Metastatic Gastric Cancer: Still More to Be Done

**DOI:** 10.3390/curroncol29090506

**Published:** 2022-09-08

**Authors:** Marzia Mare, Lorenzo Memeo, Cristina Colarossi, Dario Giuffrida

**Affiliations:** 1 Clinical Oncology Unit, Department of Experimental Oncology, Mediterranean Institute of Oncology, 95029 Catania, Italy; 2 Pathology Unit, Department of Experimental Oncology, Mediterranean Institute of Oncology, 95029 Catania, Italy

**Keywords:** metastatic gastric cancer, third line therapy, chemotherapy

## Abstract

In recent years, advances of anticancer and supportive therapies have determined a gradual improvement in survival rates and patients’ general conditions in metastatic gastric cancer (mGC), allowing them to receive further treatments. The choice of treatment is driven by performance status, age, stage of disease, number of metastatic sites and time from the first to third line of treatment. Targets such as microsatellite instability, PD-L1 expression, and HER2 overexpression or amplification may be addressed to personalise treatment and prolong survival. Despite a growing number of third line options that have provided clinicians with greater opportunities to customise treatments, up to date few agents have been demonstrated as effective after two standard lines for mGC; for these reasons, chemotherapy, immunotherapy, and targeted therapy were all widely investigated in both phase II and phase III studies. Overall, TAS-102, apatinib, regorafenib, nilotinib, trastuzumab, and pembrolizumab were demonstrated to be valid options in the third line scenario for mGC patient refractory to at least two lines of therapy. A multimodal approach based on chemotherapy, immunotherapy, targeted agents, a personalised nutritional programme as well as the research of new predictive biomarkers may pave the way to new strategies to identify the best treatment for each patient.

## 1. Introduction

Gastric cancer (GC) is the fifth most common neoplasm worldwide and the fourth cause of cancer-related mortality, according to GLOBOCAN data [1,2].

Incidence of GC varies worldwide and is largely related to carcinogenic action of *Helicobacter pylori*, cigarette smoking and first-degree family history [3,4,5].

The highest rates have been observed in Eastern Asia, Eastern Europe, and Latin America. In Europe GC represents 4% of all cancers, with a declining incidence over time for both sexes. A similar trend has also been observed in higher-prevalence nations. Nevertheless, to date GC continues to be a considerable health problem [3,6,7].

In Europe only 10 to 15% of GCs are diagnosed as “early-GC” [8]. More than half of GC patients present with locally advanced or metastatic disease [6,9].

For metastatic patients with good performance status (PS), systemic chemotherapy (CT) combining a fluoropyrimidine with a platinum agent has shown to improve overall survival (OS) when compared with best supportive care (BSC) and single-agent CT and at present it represents the standard of treatment in a first line setting [10,11,12,13].

In the case of epidermal growth factor receptor 2 (HER-2) positivity, the association of trastuzumab to CT has improved therapeutic efficacy [14,15].

After failure of first line therapy, irinotecan-based or taxane-based CTs are mostly used as standard treatment. Ramucirumab, a vascular endothelial growth factor receptor (VEGFR) monoclonal antibody, when used in combination with paclitaxel in this setting of relapsed patients, was shown to be more effective. However, the OS advantage is generally modest if compared with BSC, therefore the prognosis of advanced/metastatic GC patients to date remains poor [3,16].

Recently, new agents have been added to the therapeutic armamentarium against GC. The introduction of new CTs and targeted agents with a more favourable efficacy and toxicity profile have allowed many patients, in progression under second line treatments, to be still eligible for further therapies. Available third line options include irinotecan, taxanes, TAS-102, tyrosine kinase inhibitors, immune-checkpoint inhibitors (CPIs).

This mini-review aims to explore and summarise data from the key phase II and phase III clinical trials on the current third- and late line treatments for metastatic GC.

## 2. Efficacy and Safety of Third Line Treatments for Metastatic Gastric Cancer

In recent years advances of anticancer and supportive therapies have determined a gradual improvement in both survival rates and patients’ general condition, allowing patients to have further treatment chances [17].

**Figure 1:**
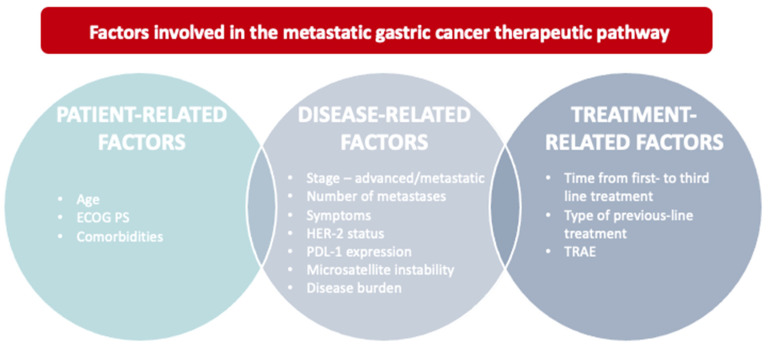
The therapeutic pathway of advanced disease in metastatic gastric cancer should derive from the evaluation of factors related both to patient (age, PS, comorbidities), and disease-related factors (stage—locally advanced vs. metastatic, presence or absence of symptoms and disease burden) as well as factors related to the therapy itself (type of drugs used in the first line with the related toxicities).

The choice of treatments is driven by factors influencing the outcomes of these patients, such as PS, age and stage of the disease, number of metastatic sites and time from the first line to third line of treatment (Figure 1) [17].

Targets such as microsatellite instability, PD-L1 expression, and human epidermal growth factor receptor-2 (HER2) overexpression or amplification may be addressed with the aim of personalising treatment and prolonging survival [18]. Unfortunately, at present few effective agents are available after two standard lines for mGC [6].

Third line options include cytotoxic agents (irinotecan, taxanes and TAS-102), immunotherapy (nivolumab, pembrolizumab, RC48, trastuzumab deruxtecan) and targeted therapy (apatinib, regorafenib, tivantinib) (Table 1).

### 2.1. Chemotherapy

In the third line setting, in a Korean study conducted by Kang et al. in 2013, the 5-fluorouracil, leucovorin, and irinotecan (FOLFIRI) regimen as a treatment showed an objective response rate (ORR) of 9.6%, a median PFS of 2.1 months and a median OS of 5.6 months [19].

Similarly, in a 2012, third line CT treatment with irinotecan or docetaxel did not improve the median OS in mCG patients relapsed following a second line CT, compared with BSC [20,21].

The poor effectiveness of the FOLFIRI regimen in the third line treatment was further confirmed in a recent observational study from Roviello et al. with patients with mGC/GEJC after progression of a previous ramucirumab-based second line therapy showing a median PFS of 52 days and a median OS of 117 days [22].

Even the addition of everolimus to paclitaxel in third line setting failed to improve the outcomes of GC/GEJC patients, despite some activity and lesser PS deterioration being seen in the taxane pre-treated group in a study published in 2020 by Lorenzen et al. [23]. Therefore, taxanes and FOLFIRI as third line CTs should be considered only when other options are not feasible [6,11].

Nevertheless, in this disappointing scenario, third line treatment with trifluridine/tipiracil (TAS-102), an oral combination of a fluoropyrimidine (trifluridine, TFT) and a thymidine phosphorylase inhibitor (tipiracil hydrochloride, TPI) showed promising survival benefits in patients with mGC [24].

TAS-102 anticancer activity is mainly exerted through incorporation within the replicating DNA strands that leads to inhibition of tumour cell proliferation. In addition, trifluridine inhibits thymidine synthetase (TS), while tipiracil prevents the degradation of trifluridine by inhibiting thymidine phosphorylase (TP), thus increasing its availability [25]. Efficacy of TAS-102 plus BSC vs. placebo plus BSC was demonstrated in the TAGS trial, a phase III randomised, double-blind, placebo-controlled study on a cohort of 507 pre-treated patients with advanced/metastatic GC [26].

Patients in the TAS-102 arm had a significant gain in OS, with a 31% reduction in the risk of death (median OS 5.7 months vs. 3.6 months, hazard ratio (HR) 0.69 (95%CI 0.56–0.85), *p* = 0.00058), if compared with those in the placebo arm. The survival efficacy was sustained regardless of prognostic factors, such as ECOG PS, number of previous treatment lines, HER2 status, number of metastatic sites and age [26].

Despite a relatively low response rate (ORR 4%), patients had 44% disease control (DCR) with an acceptable toxicity [25].

Based on the evidence from a TAGS study, recently TAS-102 received Food and Drug Administration (FDA) approval for the treatment of GC after at least two lines of CT including fluoropyrimidines, platinum compounds, taxanes or irinotecan and, if appropriate, anti-HER2 therapy [24]. To date, this drug is available in countries with reimbursement [25].

### 2.2. Immunotherapy

So far, the most promising immunotherapeutic target in GC patients is the programmed cell death receptor 1 (PD-1)/programmed cell death ligand 1 (PD-L1) checkpoint axis [27].

Among the monoclonal antibodies targeting PD-1/PD-L1, nivolumab, avelumab, pembrolizumab was the most investigated in the third line setting.

The role of nivolumab, a fully human IgG4 monoclonal antibody, was evaluated in a large phase-III study (ATTRACTION-02) on a cohort of 493 Asian patients with advanced GC/GEJC treated with at least two CT regimens, regardless of the PD-L1 status [28,29].

ATTRACTION-02 demonstrated a significantly better median OS in patients given nivolumab, compared with those given placebo (5.3 months vs. 4.1 months), with 26.2% of them still alive after a year of treatment, vs. 10.9% in the placebo group. The long-term efficacy and the survival benefit of treatment beyond progression were further confirmed after a 3-year follow-up [30]. The results of ATTRACTION-02 allowed nivolumab to be approved as a third line monotherapy for patients with unresectable advanced or recurrent GC/GEJC in Japan. However, due to the Asiatic-only population recruited for this study, approval was not yet been extended to Europe [29,31].

The efficacy and safety of pembrolizumab, a humanised IgG4 monoclonal antibody against PD-1, was investigated in a phase II, open-label, single-arm study (KEYNOTE-059) on a cohort of 259 patients with advanced GC who had previously received at least two therapy lines, irrespective of PD-L1 expression [32].

Pembrolizumab treatment determined durable responses (8.4 (1.6+ to 17.3+) months), with a longer duration in PD-L1+ patients (16.3 (1.6+ to 17.3+) months). ORR was higher as well in PD-L1+ patients than PD-L1− (15.5% vs. 6.4%, median OS 5.8 vs. 4.6 months) [32].

Data from KEYNOTE-059 led to the FDA approval of pembrolizumab as a third line treatment for progressive or previously treated patients with recurrent locally advanced or metastatic gastric or gastroesophageal junction cancer with PD-L1 CPS 1 expression. [33].

The multicentre, randomised, open-label, phase III JAVELIN Gastric 300 trial was published in 2018, to explore the efficacy and safety of avelumab, a human anti-PD-L1 IgG1 monoclonal antibody approved for other neoplasms. Avelumab was evaluated versus physician’s choice of CT in a cohort of 371 randomized patients as a third line treatment for patients with advanced GC/GEJC [34].

This study aimed to assess the superiority of avelumab in terms of OS improvement (primary endpoint), PFS and ORR [34]. Compared with commonly used CTs, avelumab showed fewer any-grade or grade 3 TRAEs, however no clinical benefit was evidenced in any of the examined subgroups, including tumour PD-L1 expression status [34].

A major issue concerning immunotherapy is that only a subset of patients achieve responses. Thus, there is the need to identify the underlying mechanisms for primary resistance to the immunotherapeutic agents.

### 2.3. Targeted Therapy

Together with immunotherapy, targeted therapy has changed the therapeutic paradigm of GC [27]. Based on the rationale that targeting angiogenesis was shown to be effective in lung, breast, kidney, liver, and colon cancer, in the last decade several studies have investigated the effects of the inhibition of vascular endothelial growth factors (VEGFs) in improving OS or PFS in mGC [35].

Regorafenib is small-molecule inhibitor of multiple tyrosine kinases. In the phase II placebo controlled INTEGRATE study on 147 recurrent or metastatic GC patients, refractory to one or two lines of chemotherapy (including prior 5FU and platinum), regorafenib demonstrated an ability to prolong PFS (2.6 versus 0.9 months in the placebo group, HR 0.40, *p* < 0.001), despite improvements in OS not being significant (5.8 versus 4.5 months, HR 0.74, *p* = 0.147) [36].

In 2013, Li et al. conducted a phase II, randomized, double-blind, placebo-controlled trial to assess the efficacy and safety of apatinib, a small molecule inhibiting VEGFR tyrosine kinase, as third- or later line treatment, and also to determine the tolerability profile of the daily dose of 850 mg, given as single or refracted dose (425 twice daily) [35].

A total of 144 patients were admitted to study and randomly assigned to three groups. Statistically significant differences resulted in both PFS and OS among the apatinib 850 mg daily, apatinib 425 mg twice daily and placebo arms (3.67, 3.20 and 1.40 months, respectively). Although the median PFS of patients on apatinib did not reach the primary endpoint of a 2.5-month increase, the significantly longer PFS in patients given apatinib translated into a longer OS (4.5 months, vs. 2.5 in the placebo group) [35].

Disease control was reached by 43% of patients, consistent with the percentages obtained in other solid tumours treated with antiangiogenic agents. The single-dose apatinib regimen was better tolerated than the twice-daily regimen [35].

In a phase III trial, mGC patients with anti-angiogenesis related AE (hypertension, proteinuria, hand-and-foot syndrome), tended to have better clinical outcomes [37].

These AEs, also reported in studies with other angiogenesis inhibitors and in patients treated with apatinib for breast cancer, have been suggested as possible surrogate markers of anti-angiogenic activity. Based on these assumptions, Liu X et al. [38] conducted a retrospective cohort analysis to investigate the correlation of anti-angiogenesis related AEs with clinical outcomes in mGC patients, using data from phase II and III studies [38].

Data emerging from this analysis indicated the association of early onset of apatinib-induced AEs with significantly prolonged median OS (169 vs. 103 days), median PFS (86.5 vs. 62 days) and a 167% increase in DCR. Therefore, hypertension, proteinuria, hand-and-foot syndrome may be considered as biomarkers for good treatment efficacy [38].

Tivantinib is a non-ATP competitive small molecule that selectively inhibits the c-Met signalling pathway. In addition, it inhibits the VEGF signalling pathway and MYC expression. Its activation has been frequently found in mGC and is associated with poor prognosis [39]. Through the VEGF signalling inhibition, tivantinib has shown anti-cancer activity both in overexpressing and in non-overexpressing c-Met GC cells.

Particularly, the combination between target therapy and immunotherapy for angiogenesis and growth pathways has gained importance in recent years [27].

In the phase III GRANITE-1 study, assessing the efficacy and safety of the oral PI3K-Akt-mTOR pathway inhibitor everolimus in 872 patients with advanced GC failing at least one or two CT lines compared with BSC, everolimus did not significantly improve OS. The safety profile observed was consistent with that observed for everolimus in other cancers [40].

Ten years later, in a randomized, double-blind phase III study, the addition of everolimus to paclitaxel in advanced GC/GEJC did not show a significant impact on OS, despite survival benefits being observed in the subgroup of patients previously treated with taxanes, who were not suitable for the combination paclitaxel-ramucirumab after failure of first line platinum therapy [23].

The effect of the antibody–drug conjugate trastuzumab deruxtecan (T-DXd) was assessed in an open-label, randomized, phase 2 trial (DESTINY-Gastric01) in patients with HER2+ advanced GC. Study results, as compared with CT, showed a significant improvement in both ORR (51% of patients in the T-DXd group vs. 14% of patients in the CT group, *p* < 0.001) and OS (median, 12.5 vs. 8.4 months). The main TRAE (treatment-related adverse events) were myelosuppression and interstitial lung disease [41].

The latest results from the DESTINY-Gastric01 trial were presented at the 2022 ASCO GI, further confirming clinical benefits in both ORR and OS of T-DXd vs. CT in patients with HER2-positive advanced GC/GEJC [42].

RC48 is a new anti-HER2 antibody agent that was investigated as a third line therapy in a single-arm phase II study published in 2021 on 125 Chinese patients with HER2-overexpressing locally advanced or metastatic GC/GEJA [43].

Results showed 24.8% ORR, despite in HER2 IHC 2 positive and FISH negative patients being lower than that of conventional HER2-positive patients (16.7% vs. 26.3%), probably due to the small sample size of the study. Median PFS and OS were 4.1 months and 7.9 months, respectively [43].

RC48 showed a good safety profile, with serious AEs occurring in 36% of patients. The most frequently reported adverse events were decreased white blood cell count (53.6%), asthenia (53.6%), hair loss (53.6%), decreased neutrophil count (52.0%), anaemia (49.6%), and increased aspartate aminotransferase level (43.2%) [43].

The subgroup analysis of trastuzumab-treated and trastuzumab-naive patients was consistent with the results seen in patients with HER2 positive locally advanced or metastatic GC/GEJC treated with trastuzumab emtansine, highlighted no significant differences in ORR, PFS and OS between groups. The encouraging data of this study suggested a potential application of RC48 as a third line treatment in patients with HER2-overexpressing AGC or gastroesophageal junction [43].

**Table 1:** Summary of the main third line treatment studies in advanced/metastatic gastric cancer. (**A**) chemotherapy; (**B**) immunotherapy; (**C**) targeted therapy.

CHEMOTHERAPY				
STUDY	DESIGN	TREATMENT	ENDPOINTS	RESULTS
**Kang et al.** [20]	Multicentre, open label, randomised phase III trial on 202 adult patients with advanced GC who have failed at least two previous CT regimens	**CT (irinotecan or docetaxel) + BSC vs. BSC alone**	**Primary**: OS	**CT + BSC vs. BSC ALONE**
				Median OS: 5.3 vs. 3.8 months BSC arm (HR, 0.657; 95% CI, 0.485–0.891; one-sided *p* 0.007)
**Kang et al.** [19]	Study conducted on 158 adult patients with m/rGC to evaluate the activity and safety of the combination CT of FOLFIRI regimen after failure of fluoropyrimidine, platinum, and taxane and to evaluate the prognostic factors for survival.	**FOLFIRI 5-[fluorouracil (5-FU), leucovorin, and irinotecan]**	**PFS, OS**	Median PFS: 2.1 months (95% CI, 1.7–2.5) Median OS: 5.6 months (95% CI, 4.7–6.5)
**Roviello et al.** [22]	Observational phase II study is to evaluate the efficacy and safety of the FOLFIRI regimen as a third-line CT for ramucirumab-pre-treated patients with metastatic gastric cancers	**FOLFIRI 5-[fluorouracil (5-FU), leucovorin, and irinotecan]**	**Primary**: Tumour response (CR, PR, SD, PD) **Secondary**: OS, PFS, safety and tumour response.	EFFICACYT umour response: o CR: 0% o PR: 11.5% o SD: 27% o PD: 61.5% Median PFS: 52 days (95% CI 42–74) Median OS: 117 days (95% CI 94–154).
				SAFETY No unexpected TRAE have been observed. At least one TRAE: 84.6% of patients At least 1 TRAE (grade > 2): 34.6% of patients treatment discontinuation due to AE: 3.8% of patients 25% dose reduction due to TRAE: 15.4% of patients
**Shitara et al.** [26]	Randomised, double-blind, multinational, placebo-controlled, phase III trial to assess the efficacy and safety of trifluridine/tipiracil in patients with mGC on 507 adult mGC patients who have failed at least two previous CT regimens	**TAS-102 (trifluridine/tipiracil) + BSC** vs. **placebo + BSC**.	**Primary**: OS **Key secondary**: PFS, safety and tolerability	**TAS-102 + BSC vs. PLACEBO + BSC**
				EFFICACY [Median OS: 5.7 vs. 3.6 months (HR 0.69 95%CI 0.56–0.85] one-sided *p* = 0.00029, two-sided *p* = 0.00058) Median PFS: 2.0 vs. 1.8 months (HR 0.57 [95%CI 0.47–0.70]; *p* < 0.0001)
				**SAFETY** Any TRAE (grade ≥ 3): 80% vs. 58% of patients SAE: 43% vs. 42% of patients One treatment-related death was reported in each group
**IMMUNOTHERAPY**				
**STUDY**	**DESIGN**	**TREATMENT**	**ENDPOINTS**	**RESULTS**
**Kang et al.** [28]	Randomised, double-blind, placebo-controlled, phase III trial (ATTRACTION-02) to investigate the efficacy and safety of nivolumab, in 493 heavily pre-treated patients unselected for PD-L1 tumour expression.		**Primary**: OS **Secondary**: PFS, ORR, DCR, DOR, BOR, maximum percentage change from baseline in the sum of diameters of target lesions.	**NIVOLUMAB vs. PLACEBO**
				Median OS: 5.26 months vs. 4.14 months (HR 0.63, 95% CI 0.51–0.78; *p* < 0.0001).
**Fuchs et al.** [32]	Open-label, single-arm, multicohort, phase 2 study (KEYNOTE-059) on 259 adult patients with advances GC/GEJC	**Pembrolizumab**	**Primary**: ORR, safety **Secondary**: DOR (all pts and pts with PD-L1–positive tumours)	EFFICACY ORR: 11.6% (95% CI 8.0–16.1%) 15.5% in pts with PD-L1+ tumours 6.9 in pts with PD-L1—tumours Median DOR: 8.4 (1.6 * to 17.3 *) months
				SAFETY TRAE (grade 3–5): 17.8% of pts Discontinuation due to TRAE: 0.8% of pts
**Bang et al.** [34]	Multicentre, international, randomised, open-label, phase III trial (JAVELIN Gastric 300) to demonstrate superiority of avelumab versus CT as a third-line in 371 adult patients with advanced GC/GEJC	**Avelumab + BSC vs. physician’s choice of CT (paclitaxel/irinotecan**)	**Primary**: OS. **Secondary**: PFS, ORR safety and tolerability	**AVELUMAB vs. CT**
				EFFICACY Median OS: 4.6 vs. 5.0 months; (HR = 1.1 [95% CI 0.9–1.4]; *p* = 0.81) Median PFS: 1.4 vs. 2.7 months; (HR = 1.73 [95% CI 1.4–2.2]; *p* > 0.99) ORR (2.2% versus 4.3%) in the avelumab versus chemotherapy arms, respectively
				SAFETY TRAEs (any grade): 48.9% vs. 74.0% of patients TRAEs (grade ≥ 3): 9.2% vs. 31.6% of patients
**TARGETED THERAPY**				
**Pavlakis et al.** [36]	Randomized, double blind phase II trial (INTEGRATE) on 152 adult patients randomly assigned at a 2-to-1 ratio and stratified by lines of prior (one or two) CT to assess the efficacy of regorafenib on advanced GC	**Regorafenib vs. placebo**	**Primary**: PFS **Secondary**: ORR (by RECIST criteria), CBS at 2 months, OS, AE	**REGORAFENIB vs. PLACEBO**
				EFFICACY Median PFS: 2.6 vs. 0.9 months (HR 0.40; 95% CI, 0.28–0.59; *p* = 0.001) ORR: 1.9% (95% CI 1–9%) vs. 0.6% (95% CI 0–11%) of patients CBS at 2 months: 46.8% vs. 9.5% Median OS: 5.8 vs. 4.5 months (HR 0.74 (95% CI, 0.5–1.08; stratified log-rank *p* 0.147).
				SAFETY SAE (at least 1): 32% vs. 18% SAE (grade 5): 2 vs. 1 pts
**Li et al.** [35]	Phase II, randomized, double-blind, placebo-controlled trial aimed to assess the efficacy and safety of daily administration of apatinib as third-line or later treatment in 144 adult patients with mGC and to determine the tolerability of the once- or a twice-daily regimen.	**Apatinib 850 mg o.d., apatinib 425 mg b.i.d. or placebo**	**Primary**: PFS **Secondary**: DCR (including CR, PR, or SD); ORR (reduction in tumor size) and QoL	EFFICACY median PFS: patients received apatinib did not reached the anticipated improvement of 2.5 months median OS: significantly longer vs. placebo (4.5 vs. 2.5 months) DCR: on average, 43% of patients given apatinib reached disease control
				SAFETY AE grade 3 to 4: hypertension (8.51% and 10.86% of patients treated with apatinib 850 mg once daily and 425 mg twice daily, respectively).
**Li et al.** [37]	Randomized double-blind, placebo-controlled, multicenter phase III trial on 273 adult patients with advanced or metastatic GC.	**Apatinib vs. placebo**	**Primary**: OS and PFS **Secondary**: ORR, DCR, QoL, and safety.	**APATINIB vs. PLACEBO**
				EFFICACY median OS: 6.5 months vs. 4.7 months (HR:0.709; 95% CI 0.537 to 0.937; *p* = 0.0149) median PFS: 2.6 months vs. 1.8 months (HR, 0.444; 95% CI, 0.331 to 0.595; *p* < 0.001) ORR: 2.84% vs. 0% (*p* = NS) DCR: 42.05% vs. 8.79% (*p* < 0.001)
				SAFETY TRAE grade 3 to 4: nonhematologic adverse events were hand-foot syndrome, proteinuria, and hypertension.
**Liu et al.** [38]	Retrospective cohort study using pooled data from two randomised double-blind, placebo-controlled clinical trials to investigate the relationship between adverse effects and antitumor efficacy of apatinib on 269 adult patients with mGC	**Apatinib vs. placebo**	**Primary**: OS **Secondary**: PFS, DCR, and ORR. Clinical outcomes were compared with and without AEs ¶ in the first 4 weeks	**CLINICAL OUTCOMES WITHOUT AES (N = 119)**
				Median OS: 103 days (IQR: 58–201 days) Median PFS: 62 days (IQR 41–121 days). Overall DCR: 82% Overall ORR: 11%
				**CLINICAL OUTCOMES WITH AES (N = 150)**
				Median OS: 169 days (IQR: 96–255 days) Median PFS: 86.5 days (IQR 57–150 days) Overall DCR: 32.77% Overall ORR: 5.04%
**Kang et al.** [39]	Multicenter, single arm open label phase II trial (ARQ 197)among 31 adult patients with mGC	**Tivantinib**	**Primary**: DCR **Secondary**: PFS, OS, and ORR.	DCR: 36.7 Median PFS: 43 days (95% CI: 29.0–92.0) ORR: 0%
				AE (grade 3–4): 43.3% of patients
**Ohtsu et al.** [40]	Double-blind phase III study (GRANITE-1) to compare the efficacy and safety of everolimus vs. BSC on 656 previously treated patients with advance GC	**Everolimus + BSC vs. placebo vs. BSC**	**Primary**: OS **Secondary**: PFS, ORR and safety	**EVEROLIMUS + BSC vs. PLACEBO vs. BSC**
				Median OS: 5.4 vs. 4.3 months (HR 0.90; 95% CI, 0.75–1.08; *p* 0.124). Median PFS: 1.7 vs. 1.4 months (HR 0.66; 95% CI, 0.56 to 0.78)
				AE (at least 1): 99.1% vs. 96.7% pts AEs leading to discontinuation: 21.5% vs. 15.8% pts
**Peng et al.** [43]	Single-arm, open-labelled, phase II trial assessing the efficacy and safety of a novel anti-HER2 therapeutic antibody RC48 in patients with HER2-overexpressing, locally advanced or metastatic GC/GEJA	**RC48**	**Primary**: ORR (CR or PR). **Secondary**: PFS, OS, DOR, TTP, DCR (CR, PR, or SD), and safety.	EFFICACY ORR: 24.8% (95% CI 17.5–33.3%). median PFS: 4.1 months (95% CI: 3.7–4.9 months) median OS: 7.9 months (95% CI: 6.7–9.9 months)
				SAFETY TRAE (grade 3–5): 56.8% pts SAEs: 36.0% of patients RC48-related SAE: decreased neutrophil count in 3.2% pts AEs resulting in dose interruption, drug suspension, or discontinuation: 10.2% pts
**Shitara et al.** [41]	Open-label, randomized, phase 2 trial, we evaluated trastuzumab deruxtecan (T-DXd) as compared with chemotherapy in 187 adult patients with HER2-positive advanced gastric cancer	**T-DXd vs.** **physician’s choice of CT**	**Primary**: ORR **Secondary**: OS, DOR, PFS, confirmed response (persisting ≥4 months), and safety.	**T-DXd vs. CT**
				EFFICACY ORR: 51% vs. 14%, *p* < 0.001 Median OS: 12.5 vs. 8.4 months (HR0.59, 95% CI 0.39–0.88; *p* = 0.01)
				SAFETY TRAE (any grade): 100% vs. 98% of pts Discontinuation due to TRAE: 15% vs. 6% Interruption due to TRAE: 62% vs. 37% Dose reduction due to TRAE: 32% and 34%

Abbreviations: BOR, best overall response; CBS, clinical benefit status CI, confidence interval; CR, complete response; CT, chemotherapy; DCR, disease control rate; DOR, duration of response; ECOG, Eastern Cooperative Oncology Group; FTD/TPI, trifluridine and tipiracil as hydrochloride; GA, gastric adenocarcinoma; GC, gastric cancer; GEJA, gastroesophageal junction adenocarcinoma; GEJC, gastroesophageal junction cancer; HR, hazard ratio; irAE, immune-related adverse events; IRC, independent review committee; mGC, metastatic gastric cancer; ORR, objective response rate; OS, overall survival; QoL, quality of life; PD-1, programmed cell death-1; PK, pharmacokinetic; PFS, progression-free survival; PR, partial response; PS, performance status; RECIST, Response Evaluation Criteria in Solid Tumors; TRAE, treatment-related adverse events; Tmab+, patients with prior trastuzumab use; Tmab-, patients without prior trastuzumab use; TTP, time to progression. (*) indicates that patients had no progressive disease at their last assessment; (¶) AE are defined as hypertension, proteinuria, or hand-foot syndrome in the first 4 weeks of treatment.

## 3. Conclusions and Future Perspectives

The growing number of third line options have provided clinicians with greater opportunities to customise treatments. According to various studies, around 20 to 90% of patients were able to continue third line or further lines of treatment. Despite there not being an internationally standardised third line to date [44], recent advances in the treatment of mGC open the door to alternative scenarios for patients undergoing a third- or later line therapy.

Overall, the drugs mentioned in this review are the most promising agents in the third line scenario, all demonstrated to be valid options for mGC patients, progressing through at least two lines of therapy.

Combination of chemotherapy, immunotherapy, and targeted agents as well as the research for new predictive biomarkers may open the door to new approaches aimed to fit the best treatment for each patient.

In this regard, two different molecular screening programmes (VIKTORY and PANGEA) both evaluating treatment options based on the expression of specific biomarkers, confirmed that the OS and PFS rates were better in those patients receiving the selected therapy according to the specific biomarker expression [45,46].

Furthermore, the role of nutritional therapy and simultaneous care are widely recognized, with oncologic treatment’s tolerance and response being better in well-nourished patients; therefore, maintaining an ideal nutritional status is essential to improve treatment benefits [47].

Current guidelines highlight the importance of a precise nutritional assessment and a good definition of oncologic programmes to identify the proper risk/benefit balance of nutritional interventions. A home parenteral nutrition approach administered even for only 1 or 2 months in mGC patients seems to be a promising strategy in mGC patients, positively correlating with a better QoL and nutritional status [47].
